# The Etiology of Reduced Muscle Mass with Surgical and Pharmacological Weight Loss and the Identification of Potential Countermeasures

**DOI:** 10.3390/nu17010132

**Published:** 2024-12-31

**Authors:** Isabella Faria, Sarah Samreen, Lauren McTaggart, Emily J. Arentson-Lantz, Andrew J. Murton

**Affiliations:** 1 Department of Surgery, University of Texas Medical Branch, Galveston, TX 77554, USA; imdefrei@utmb.edu (I.F.); sasamree@utmb.edu (S.S.); lsmctagg@utmb.edu (L.M.); 2 Department of Nutrition Sciences & Health Behavior, University of Texas Medical Branch, Galveston, TX 77554, USA; ejlantz@utmb.edu; 3 Sealy Center on Aging, University of Texas Medical Branch, Galveston, TX 77554, USA; 4 Center for Health Promotion, Performance and Rehabilitation Science, University of Texas Medical Branch, Galveston, TX 77554, USA

**Keywords:** obesity, bariatric surgery, GLP-1 receptor agonists, muscle loss, nutritional interventions, sarcopenia, muscle protein turnover

## Abstract

Obesity represents a major health crisis in the United States, significantly increasing risks for chronic diseases and generating substantial economic costs. While bariatric surgery and pharmacological interventions such as GLP-1 receptor agonists have been proven effective in achieving substantial weight loss and improving comorbid conditions, they also raise concerns about the unintended loss of fat-free mass, particularly muscle. This loss of muscle mass compromises physical functionality, quality of life, and long-term metabolic health, particularly in individuals with sarcopenic obesity or those at risk of frailty. To sustain strength, mobility, and metabolic function during weight loss interventions, the preservation of muscle mass is essential. However, current weight-loss strategies often fail to adequately address the need to maintain fat-free mass. This review explores the physiological mechanisms governing muscle mass, the impact of obesity and rapid weight loss on muscle protein turnover, and nutritional and age-based strategies that may help protect muscle during intentional weight reduction. By focusing on these critical countermeasures, this review aims to inform future clinical practice and research initiatives with the long-term goal of achieving effective weight loss through reduction in fat tissue while preserving skeletal muscle mass, enhancing health outcomes, and long-term functionality in patients undergoing significant weight reduction.

## 1. Introduction

Obesity remains a critical health concern in the United States, affecting over 40% of adults and contributing substantially to the burden of chronic diseases such as type 2 diabetes, cardiovascular disease, and certain cancers [1,2]. The economic impact of obesity is equally profound, as it leads to increased individual and system-level healthcare costs, reduced workforce productivity, and a substantial decrease in quality of life among affected individuals [3]. As a result, sustainable and effective weight-loss strategies are essential to address both the physical and economic impacts of obesity.

Bariatric surgery and pharmacological interventions, particularly glucagon-like peptide-1 (GLP-1) receptor agonists, have shown considerable effectiveness in achieving clinically significant weight loss [4]. Bariatric procedures, such as Roux-en-Y gastric bypass and sleeve gastrectomy, are the gold standard for patients with severe obesity [5]. These approaches frequently lead to sustained reductions in body mass and improvements in obesity-related comorbidities [4,5]. GLP-1 agonists, originally developed for glycemic control in type 2 diabetes, have demonstrated weight loss effects and have been increasingly utilized for this purpose, particularly in the past couple of years [4]. However, rapid weight loss promoted by both surgical and pharmacological approaches also carries the risk of inducing rapid loss of fat-free mass (FFM), particularly skeletal muscle. A recent meta-analysis highlighted that the loss of lean body mass (LBM), a surrogate marker of muscle mass, represents a significant proportion of the weight loss experienced in the first 12 months following metabolic and bariatric surgery [6]. On average, patients lose around 8 kg of LBM in the first 12 months following surgery, with about half of that loss occurring within the first 3 months before the rate of loss tapers [6,7].

Preserving muscle mass during weight loss is essential for maintaining a high quality of life and preventing reductions in strength, mobility, and metabolic health [8]. Furthermore, muscle tissue plays a key role in energy expenditure, glucose homeostasis, and physical functionality. Loss of muscle mass can impair these functions, potentially compromising long-term weight maintenance and overall health outcomes [8]. Muscle loss is particularly concerning for individuals undergoing intentional weight reduction who may already face risks of frailty and diminished functional capacity, especially in the elderly and among populations with sarcopenic obesity [9]. The difficulty of preserving fat-free mass during significant weight reduction underscores a crucial need within the current treatment paradigm.

In response to these challenges, this review will examine the physiological and nutritional regulators of muscle mass, with a particular focus on how obesity and weight loss affect muscle protein turnover, a key determinant of muscle mass. Nutritional strategies that can decrease the loss of muscle mass and practical recommendations to support the preservation of muscle function and metabolic health will be identified. Lastly, future avenues for research and potential changes in clinical practice that could promote the reduction in body fat while maintaining fat-free mass in patients undergoing weight loss will be discussed.

## 2. The Essential Role of Nutrition in the Regulation of Muscle Mass

Skeletal muscle, by virtue of its contractile function, is comprised of multinucleated fibers containing myofibrillar proteins arranged in register to form organized contractile units [10]. Damage to the contractile apparatus, as seen over time or with overexertion [11], necessitates a constant replacement cycle of the myofibrillar apparatus, with approximately 1.2% of the total muscle protein pool turned over daily in healthy young adults [12]. When a persistent imbalance between the rates at which myofibrillar proteins are synthesized and degraded exists, a reciprocal expansion or shrinkage of the myofibrillar protein pool occurs. By extension, a loss of muscle mass occurs when the net rate at which proteins are degraded consistently exceeds the rates at which they are replaced. Thus, given the positive relationship between muscle volume and maximal force production [13], the maintenance of muscle protein homeostasis is essential for the preservation of both muscle mass and contractile function and a focus on interventions aimed at preventing or slowing unintended muscle loss.

**Figure 1:**
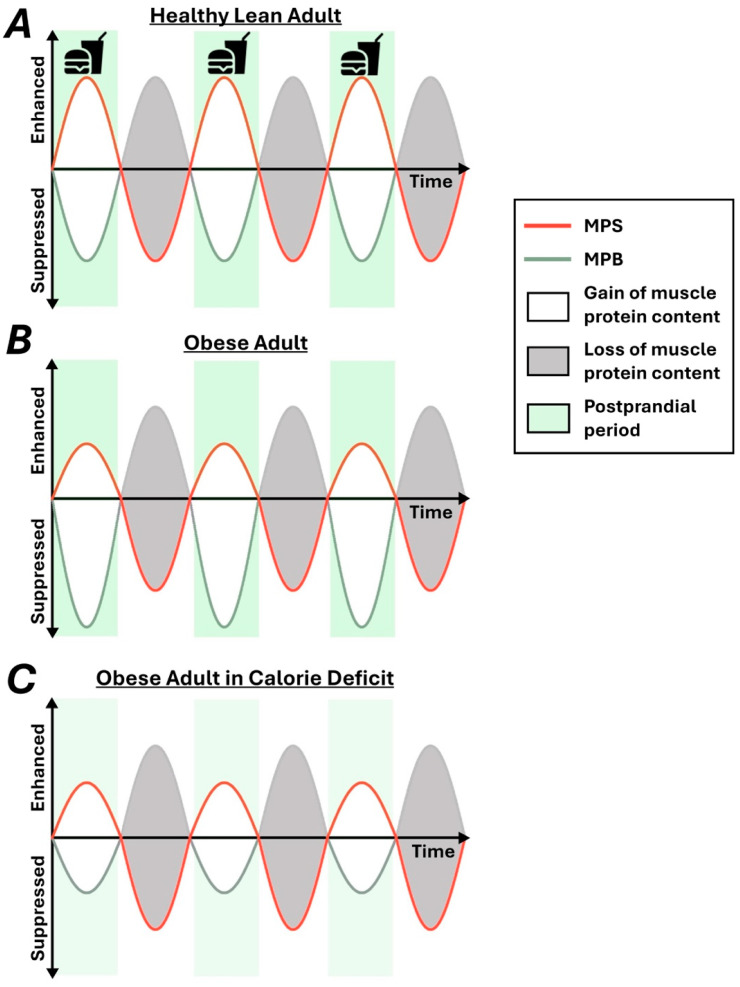
Diagrammatic representation of the impact of nutrition and fasting on muscle protein turnover. Kinetic changes in muscle protein synthesis (MPS) and muscle protein breakdown (MPB) in healthy young lean (**A**), obese (**B**), and obese individuals undergoing weight loss treatment (**C**). Net protein balance (the difference between the gain and loss of muscle protein content) is largely neutral in lean and obese subjects (**A**,**B**) but declines with reduced food intake (**C**).

The opposing processes of muscle protein synthesis and breakdown are in a constant state of flux, responding to changing nutritional, contractile, and hormonal cues throughout each day [14,15]. The increased availability of free amino acids in the circulation following food consumption acts as a particularly potent trigger for the stimulation of muscle protein synthesis [16]. In healthy young adults, the hyperaminoacidemia seen following the consumption of even a modest bolus of essential amino acids (5 g) elicits an approximate doubling in the production rate of myofibrillar proteins across 3 h post-consumption [17]. Conversely, hyperinsulinemia, as seen following food consumption, equally exerts a positive influence on muscle protein accretion by simultaneously suppressing muscle proteolysis [15]. Collectively, the stimulation of muscle protein synthesis concomitant to the suppression of muscle protein breakdown under postprandial conditions offsets the loss of muscle protein content during periods of fasting (Figure 1). Consumption of a mixed meal containing protein, by increasing amino acid concentrations in the circulation and inducing endogenous insulin secretion [18], acts as an instrumental regulator of muscle protein turnover and reinforces the rationale for the focus on maintaining adequate protein intake in obese individuals undergoing pronounced calorie-restricted diets.

Crucially, the stimulatory effects of amino acids on muscle protein synthesis differ based on the amino acids involved. Comparing the effect of an oral bolus of essential amino acids on muscle protein synthesis with that of a bolus of mixed amino acids containing an equivalent quantity of the same essential amino acids, Volpi and colleagues demonstrated that the stimulatory effect of the amino acids on muscle protein synthesis occurred predominantly in response to the essential amino acids alone [19]. Moreover, subsequent experiments have shown that the branched-chain amino acid leucine is solely sufficient to stimulate muscle protein synthesis [20], possessing a unique capability to activate muscle cellular signaling pathways that are instrumental in the induction of protein translation [21]. Thus, both the quantity and quality of protein consumed appear to be important considerations for individuals who are abiding by a calorie-restricted diet and want to preserve muscle mass.

## 3. Obesity-Induced Derangements in Muscle Protein Metabolism

Obesity has several impacts on muscle protein metabolism, creating challenges for protecting muscle mass in individuals embarking on rapid weight loss. Firstly, the presence of insulin resistance in obese individuals is common, with ~60% of individuals with a body mass index > 35 kg·m^−2^ demonstrating some impairment in insulin sensitivity [22]. Crucially, insulin-dependent hyperemia plays an essential role in skeletal muscle nutrient delivery, stimulating vasodilatation and increasing blood flow towards the microvasculature that supplies the individual muscle fibers (see [23]), delivering nutrients, including circulating amino acids for cellular protein synthesis. Reinforcing the permissive role of vasodilatation in the stimulation of muscle protein synthesis by circulatory factors, sodium nitroprusside, a potent vasodilator, has been shown to enhance the protein synthetic response to insulin infusion in older adults [24]. Thus, obesity-related insulin resistance could act as a barrier to the maintenance of net muscle protein balance and, by extension, muscle mass, but this remains to be empirically determined. Given that both bariatric surgery and GLP-1 administration are associated with improvements in insulin sensitivity [25,26], the contribution that blunted hyperemia post-food consumption has on muscle protein synthesis following the induction of reduced calorie intake is currently unclear and should be the focus of future work.

Obese individuals may also have an impaired ability to successfully utilize dietary amino acids for the synthesis of new muscle contractile proteins and, thereby, a reduced ability to offset the habitual breakdown of the contractile apparatus. Examining the impact of obesity on muscle protein metabolism, we reported that the ability of older obese men to successfully utilize intravenously administered mixed amino acids to synthesize myofibrillar proteins was significantly diminished compared to their age-matched lean counterparts [27]. Moreover, given that cell signaling pathways instrumental in promoting the metabolic actions of insulin and amino acids in muscle were activated to equivalent degrees in lean and obese individuals [27], our work suggests alternative intracellular mechanisms underpin the blunted anabolic response to exogenous amino acids in the obese. While other groups have also reported the existence of muscle anabolic resistance to exogenous amino acids in the obese [28,29,30], it has not been universally observed [31,32,33], and the mechanisms underlying this phenomenon remain largely unresolved. Nevertheless, for the individual seeking weight loss treatment, the purported reduced ability of dietary amino acids to maintain an adequate rate of muscle contractile protein renewal is concerning. Based on these findings, it is likely that the quantity, quality, and timing of dietary protein intake necessary to maximally stimulate muscle protein synthesis differ in the obese compared to the wider population upon which dietary guidelines are largely based.

Despite the presence of metabolic changes that would be expected to create challenges to the maintenance of muscle mass in the obese, skeletal muscle mass is often greater than that seen in lean individuals of similar height and age [34]. Historically, this has been thought to be due to the additional contractile work required for locomotion in individuals carrying excess body weight [35], where weight-bearing contraction is a known potent stimulator of muscle hypertrophy. While a plausible explanation, we have observed the existence of an alternative mechanism that may explain the preserved muscle mass in the obese in the face of metabolic insults. Examining the impact of obesity on muscle protein metabolism, we observed that increased adiposity was associated with the enhanced suppression of muscle proteolysis under hyperinsulinemia and was sufficient to counter the impact of diminished protein synthesis rates following amino acid provision on muscle net protein content [27]. While our observations of reduced muscle protein turnover in the obese could lead to a failure to replace damaged contractile proteins in a timely fashion, explaining the reduction in muscle quality reported by others [36,37,38], it should be noted that declines in muscle maximal isometric force and fatigability were not noted in our subjects [27]. While the obesity-induced impediment of muscle proteolysis appears to confer some degree of protection against the loss of muscle mass, this is likely lost rapidly when individuals consume a calorically restricted diet. As early as 24 h from the onset of a fast, markers synonymous with the enhancement of muscle proteolysis are increased in rodents [39]. While direct evidence that calorie-restricted diets enhance muscle protein breakdown rates in humans remains elusive, the failure to observe a deterioration of postprandial muscle protein synthesis rates in individuals undergoing prolonged calorie-restricted diets who are simultaneously experiencing the loss of appendicular lean mass suggests that muscle proteolysis rates are accelerated during weight loss [40]. Based on these findings, the study of nutritional, pharmacological, and exercise countermeasures that can limit muscle proteolysis during calorie-restricted diets would be advantageous in the quest to protect muscle mass and function.

## 4. Nutritional Strategies to Blunt Muscle Loss During Pharmacological and Surgery-Induced Weight Loss

Nutritional support following bariatric surgery, particularly careful tracking of dietary protein intake, is the frontline measure to provide both the amino acid pool necessary to preserve fat-free mass during rapid weight loss as well as promote tissue healing. Dietary counseling from a registered dietitian is critical for nutrition and meal planning guidance before and after surgery for patients to be successful in meeting weight loss goals [41] and energy and macronutrient needs. However, one of the greatest challenges in providing nutritional support for dietary protein intake is the conflicting information and lack of evidence to establish dietary protein needs following bariatric surgery [42]. A recent joint summary from the leading medical organizations that provide best clinical practices for perioperative nutrition and metabolic care of bariatric patients provided input from different sets of dietary protein guidelines ranging from (i) a minimum of 46 g/day for women and 56 g/day for men or 0.8–1.2 g/kg body weight/day for weight maintenance or >1.2 g/kg/body weight/day for active weight loss [43], (ii) an average of 60–120 g daily [44], or (iii) have not yet defined dietary protein needs [45,46]. The summary recommendation was a minimal protein intake of 60 g/d and up to 1.5 g/kg of ideal weight per day with higher amounts of protein, up to 2.1 g/kg ideal weight per day, to be assessed on an individualized basis. However, this evidence was graded as “D”, meaning it is primarily based on expert opinion. For perspective, 46 g/day and 56 g/day of protein are sufficient to meet the recommended dietary allowance (RDA) of 0.8 g/kg/day, which is the minimum amount of protein needed to prevent deficiency for a 57.5 kg and 70 kg healthy adult, respectively, and are unlikely to provide a sufficient amount of dietary protein to preserve the loss of lean mass that occurs in a perioperative bariatric patient. It is important to consider that these recommendations are based on hypoalbuminemia or lean tissue mass retention [47] rather than a direct measure of nitrogen balance, protein requirements, or muscle anabolism. A recent nitrogen balance study of morbidly obese patients before, as well as 3 and 12 months following, bariatric surgery indicated increased protein requirements in severe and morbidly obese patients before and after the procedure [28]. The paucity of studies that directly assess dietary protein needs for bariatric patients presents a challenge for dietitians and other health professionals seeking to provide evidence-based nutrition guidance to prevent post-procedure loss of lean mass as well as support immune function and wound healing.

While several randomized-controlled trials have examined the efficacy of high-protein diets or protein supplementation to preserve fat-free mass (FFM) following bariatric surgery, the data are equivocal. A recent meta-analysis and systematic review that included eight randomized-controlled trials evaluated the effects of dietary protein intervention (high-protein diet or protein supplementation) on body composition following bariatric surgery [48]. The authors observed that overall, there were no significant differences in postoperative FFM in participants in the intervention groups that were counseled on a high-protein diet or received a protein supplement, although increasing dietary protein intake contributed to greater weight loss (4.95 kg) and fat mass loss (7.64 kg). Notably, fat-free mass was preserved following bariatric surgery in two studies where laparoscopic adjustable gastric banding patients achieved 1.2 g/kg/day [49] or 2 g/kg ideal body weight/day of dietary protein, and in another study that assessed protein intake and FFM status in sleeve gastrectomy patients, dietary protein intake was a small but significant indicator of FFM following surgery [50]. Achieving >1.0 g/kg per day of dietary protein to preserve FFM during weight loss is supported by the literature on non-surgical weight loss in adults [51] and is likely a more ideal target protein intake level than is currently being recommended for most bariatric patients.

In addition to the amount of daily protein that is consumed, considering the quality, timing, and distribution of protein at each meal [52,53,54], rather than simply 24 h intake [55], is likely important to ensure sufficient stimulation of tissue anabolism by exogenous amino acids in weight loss patients. Foods containing protein are typically classified as sources of complete or incomplete proteins based on their essential amino acid (EAA) composition and capacity to meet human EAA needs. Animal-based proteins are recognized as a higher quality protein based on the comprehensive EAA profiles and high ratings on the Digestible Indispensable Amino Acid Score (DIAAS), reflecting their greater metabolic availability and potential benefits for muscle health [56,57]. Conversely, plant-based proteins often are composed of suboptimal quantities of one or more EAAs, resulting in lower DIAAS ratings, and are considered lower-quality proteins [57]. Consuming 25–30 g of complete protein per meal likely delivers an adequate EAA profile and quantity to effectively promote muscle protein synthesis (MPS) for healthy adults [53,54,55]. Conversely, a much greater quantity and combination of incomplete, plant-based proteins are typically required to provide a complete amino acid profile and similar anabolic stimulus [58,59], making it difficult to consume adequate amounts from solely plant-based sources when portion sizes are restricted or protein-induced satiety is evident [60]. Because an increased food volume is likely to present a challenge for bariatric patients in particular, consuming moderate amounts of high-quality protein at each meal is likely crucial for meeting protein intake goals and inducing a sufficient stimulus to promote muscle protein anabolism.

Although consuming adequate amounts of dietary protein is important for preserving FFM and promoting weight loss, data from observational studies indicate bariatric patients fail to achieve this, with most consuming 40–80 g/day [61,62,63,64]. In randomized controlled trials where patients received protein supplementation or regular dietary counseling to maintain higher protein intake, compliance with the intervention recommendations was low, and many participants did not achieve the recommended protein intake [50,65,66]. By design, the reduced gastric capacity contributes to reduced food volume at each meal, and patients describe having lower hunger levels and more postprandial fullness, especially for the first 12 months following surgery. Additionally, protein consumption may contribute to a greater level of satiety and reduce the desire to eat again in part by stimulating the release of anorexigenic gut hormones, including glucagon-like peptide-1, peptide YY, and cholecystokinin [67]. To address the issues of reduced food protein intake due to increased satiety, some groups have evaluated the efficacy of supplementing diets with branched-chain amino acids to stimulate muscle anabolism. Supplementing gastric sleeve patients with 40 g whey protein, 2000 IU Vitamin D, 40 mg of leucine, 20 mg valine, and 20 mg isoleucine resulted in significantly less loss of FFM in the first 30 days following the procedure compared to patients receiving protein supplementation alone [68]. Similarly, RYGB patients supplemented with a product based on short peptides lost 50% less FFM when compared to those supplemented with either hydroxy methylbutyrate (HMB), a metabolite of leucine, or counseled to consume 80–90 g of protein [69]. Together, these data suggest that the use of a targeted peptide or BCAA supplementation is useful to overcome the anabolic resistance associated with obesity. Additionally, it may help obese patients consume appropriate amounts of dietary protein during managed weight loss. However, evidence-based guidelines for the quantity, quality, and timing of dietary protein intake to stave off muscle loss in patients undergoing managed weight loss are currently lacking and should be the focus of future efforts.

An alternative strategy to preserve muscle mass in weight loss patients is by encouraging tailored exercise. Physical activity, particularly resistance exercise, stimulates muscle anabolism and has been investigated as an interventional strategy to preserve FFM following bariatric surgery. While resistance exercise [70,71,72], or a combination of resistance and cardio exercise [73,74,75], reduces the decline in muscle strength observed following bariatric surgery and improves muscle architecture [76], participants are still shown to lose a significant amount of FFM [77]. As pointed out by a recent systematic review on resistance exercise following bariatric surgery [77], relatively few studies recorded protein or supplement intake; therefore, it is possible the efficacy of resistance exercise to preserve FFM may be impaired by the availability of dietary amino acids to support the anabolic response of skeletal muscle to contractile activity. Further studies are needed to understand the synergistic effect of exercise and dietary protein to support fat-free mass preservation in weight loss patients.

## 5. The Compounding Effect of Age on the Maintenance of Muscle Mass During Intentional Weight Loss

The compounding effects of an aging population and increasing incidence of obesity [78] present unique challenges for the safe and effective application of contemporary weight loss strategies. Despite this, there remains a dearth of information on the effects of bariatric surgery or GLP-1-induced weight loss on the maintenance of muscle mass and function in older individuals. Of concern, several age-related metabolic and physiological changes occur in later life that likely place these patients at heightened risk for the development of sarcopenia and associated comorbidities. As early as the mid-forties, the muscle mass and strength of healthy, recreationally active individuals begin to decline, accelerating in the final decades of life and often resulting in frailty development [79,80]. Notably, the onset of physical impairment in obese individuals may occur at an earlier age even despite them possessing a perceived greater musculature than their non-obese counterparts. Woo and colleagues, examining the relationship between BMI, body composition, and physical function in older adults, demonstrated an increased number of impairments in instrumental activities of living and a decreased walking performance in obese vs. lean individuals, despite the former having a greater appendicular lean mass [81]. Indeed, the authors found an association between an increase in fat mass and a decline in physical function independent of appendicular muscle mass, observations that are in keeping with the reports of others [82]. Collectively, these findings support longitudinal reports demonstrating an increased risk of frailty in obese populations over time [83,84,85,86].

**Figure 2:**
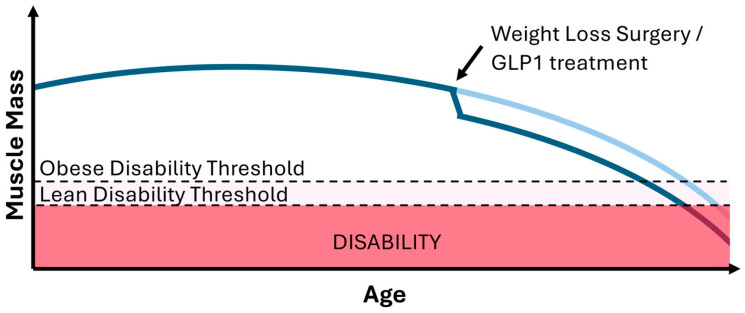
Theoretical impact of weight-loss-induced muscle catabolism on the acceleration of age-related disability. The loss of muscle mass with weight loss surgery or GLP-1 treatment is common and concerning for those who fail to maintain adequate weight loss, particularly in later life, where the regain of muscle mass is challenging.

Given that the loss of muscle mass is common with rapid weight loss [87], and weight regain in these individuals remains a concern, there is the potential for the early onset of age-related frailty and loss of independence (Figure 2). Stenholm and colleagues, surveying more than 2000 Finnish individuals, observed that those who self-reported a previous history of obesity but were currently non-obese had an increased risk of walking limitation compared to individuals who had no prior history of obesity [88]. The negative effect of a previous history of obesity on physical limitations was independent of effects of age, gender, socioeconomic, and health behavior factors (e.g., smoking), although the impact of obesity-related diseases could not be excluded. From their work, it is clear that a prior history of obesity has a burden on physical function in later life independent of current weight status, but the mechanism(s) driving this effect is currently unclear. Thus, understanding the metabolic events underpinning the loss of muscle mass in individuals undergoing managed weight loss and developing strategies that can promote fat mass loss while minimizing the reciprocal loss of muscle mass is of critical importance to the older population.

While the causes of sarcopenia are multifaceted, dysregulated muscle protein metabolism in response to dietary intake is thought to play a major contributory role. By utilizing escalating oral doses of mixed amino acids, Cuthbertson and colleagues were able to demonstrate that the ability of exogenous amino acids to stimulate muscle protein synthesis is profoundly blunted in the elderly when compared to young adults. More importantly, this cannot be overcome by simply increasing amino acid availability alone [17]. Similar observations of this phenomenon have also been confirmed by various other groups studying the biological hallmarks of sarcopenia [89,90]. This is compounded by additional observations detailing the inability of insulin to suppress muscle proteolysis in the elderly [91], collectively contributing to the habitual loss of muscle protein content over time and representing a key driver of sarcopenia. Concerningly, these findings suggest that a nutritional approach to stave off sarcopenia in obese elders undergoing weight loss could be challenging.

An additional hurdle for the aging obese individual is the likelihood of sedentary behavior and its impact on muscle metabolic health. While sedentary behavior is commonly associated with the development of unintended weight gain [92], prolonged obesity in the elderly can help to reinforce sedentary behavior, with impaired joint pathology making ambulation painful [93] and age-associated declines in muscle mass creating challenges for locomotion [94]. Furthermore, sedentary behavior can additionally impair the ability of dietary amino acids to stimulate muscle protein anabolism beyond the effects of old age alone. Indeed, while we have shown that even a short bout of limb immobilization in the young adult blunts the synthesis of muscle proteins under postprandial conditions [95], a modest decline in activity has been shown sufficient to induce similar effects in the elderly [96]. Breen and colleagues, examining the impact of an 80% reduction in daily step count for 2 weeks on protein metabolism in elderly individuals, reported a 26% decline in the protein synthetic response to a 25 g oral protein bolus and a ~3.9% decline in leg muscle mass over the period [96]. Collectively, these findings demonstrate the crucial role of encouraging individuals undergoing bariatric surgery or GLP-1-mediated weight loss to incorporate physical activity into their daily routine to maintain the anabolic potential of nutrition.

Lastly, it should be noted that significant overlap exists in the muscle metabolic derangements seen in muscle with advancing age and obesity. Both are characterized by a sedentary lifestyle [97,98], the development of insulin resistance [22,99], low-grade inflammation [100,101], and failure of exogenous amino acids to efficiently stimulate muscle protein synthesis [17,27]. It appears as though aging and obesity are independent risk factors for impaired muscle protein metabolism. However, it is not certain whether aging and obesity work in a synergistic fashion, resulting in accelerated muscle “metabolic aging”. Given obesity is a modifiable risk factor, uncoupling its impact from aging will be imperative to optimize the response to surgical and pharmacological weight loss strategies in older adults.

## 6. Conclusions

Preserving muscle mass during significant weight loss is a critical yet often overlooked component of obesity management. Bariatric surgery and pharmacological interventions, such as GLP-1 receptor agonists, effectively reduce body weight and improve obesity-related comorbidities but frequently result in substantial loss of lean body mass, particularly in the early stages of treatment. Muscle mass plays a pivotal role in metabolic health and physical functionality. Its loss can lead to diminished strength, mobility, and long-term weight maintenance, making its preservation essential for sustainable health outcomes.

Addressing this challenge requires a nuanced understanding of the physiological, nutritional, and behavioral factors that influence muscle protein turnover during rapid weight loss. Nutritional strategies, such as adequate dietary protein intake, tailored amino acid supplementation, and careful timing, as well as consideration of the quality of protein consumed, show promise in mitigating muscle loss. However, existing guidelines for dietary protein needs following bariatric surgery or GLP-1 treatment remain inconsistent and lack the robust evidence required to establish precise recommendations. Thus, this underscores the need for further research. Optimizing muscle preservation through personalized approaches and integrating resistance exercise into post-surgical care protocols could enhance outcomes for bariatric patients, particularly among older individuals and those with sarcopenic obesity.

Moving forward, collaborative efforts between clinicians, dietitians, and researchers are essential to develop evidence-based interventions that support the dual goals of reducing fat mass while maintaining fat-free mass and improving quality of life. Developing personalized, targeted interventions will not only improve physical functionality and metabolic health but also enhance quality of life and long-term weight-loss sustainability. As surgical and pharmacological weight-loss strategies gain traction, addressing the preservation of fat-free mass must become a central principle of obesity treatment paradigms to maximize patient outcomes.
